# Gene clusters based on OLIG2 and CD276 could distinguish molecular profiling in glioblastoma

**DOI:** 10.1186/s12967-021-03083-y

**Published:** 2021-09-26

**Authors:** Minjie Fu, Jinsen Zhang, Weifeng Li, Shan He, Jingwen Zhang, Daniel Tennant, Wei Hua, Ying Mao

**Affiliations:** 1 Department of Neurosurgery, Huashan Hospital, Fudan University, Shanghai, China; 2 Institute of Neurosurgery, Fudan University, Shanghai, China; 3 Shanghai Key Laboratory of Brain Function Restoration and Neural Regeneration, Shanghai, China; 4 School of Computer Science, University of Birmingham, Edgartown, UK; 5 Institute of Metabolism and Systems Research, University of Birmingham, Edgartown, UK

**Keywords:** Glioblastoma, Molecular subtype, OLIG2, CD276, Random forest

## Abstract

**Background:**

The molecular profiling of glioblastoma (GBM) based on transcriptomic analysis could provide precise treatment and prognosis. However, current subtyping (classic, mesenchymal, neural, proneural) is time-consuming and cost-intensive hindering its clinical application. A simple and efficient method for classification was imperative.

**Methods:**

In this study, to simplify GBM subtyping more efficiently, we applied a random forest algorithm to conduct 26 genes as a cluster featured with hub genes, OLIG2 and CD276. Functional enrichment analysis and Protein–protein interaction were performed using the genes in this gene cluster. The classification efficiency of the gene cluster was validated by WGCNA and LASSO algorithms, and tested in GSE84010 and Gravandeel’s GBM datasets.

**Results:**

The gene cluster (n = 26) could distinguish mesenchymal and proneural excellently (AUC = 0.92), which could be validated by multiple algorithms (WGCNA, LASSO) and datasets (GSE84010 and Gravandeel’s GBM dataset). The gene cluster could be functionally enriched in DNA elements and T cell associated pathways. Additionally, five genes in the signature could predict the prognosis well (*p* = 0.0051 for training cohort, *p* = 0.065 for test cohort).

**Conclusions:**

Our study proved the accuracy and efficiency of random forest classifier for GBM subtyping, which could provide a convenient and efficient method for subtyping Proneural and Mesenchymal GBM.

**Supplementary Information:**

The online version contains supplementary material available at 10.1186/s12967-021-03083-y.

## Introduction

Glioblastoma (GBM) is the most common malignant primary brain tumor. The past decades have witnessed considerable advances in neurosurgery and radio-chemotherapy but limited survival benefits [1]. Many efforts were made to set several classification schemes and capture this variability by using gene expression data to identify more homogeneous sub-categories for prognosis and drug sensitivity. Molecular signatures such as 1p/19q co-deletion, MGMT methylation, IDH mutation, TERT promoter mutation and H3F3A mutation have advanced the integrative subtype profiling of gliomas [2]. The most used classification scheme was proposed by Verhaak et al., which divides GBMs into Proneural, Classical, Neural, and Mesenchymal types based on gene expression measured with mRNA microarrays from TCGA [3]. Generally, the proneural GBM patients are young and characterized with a good prognosis, while the mesenchymal subtype has the worst prognosis. CGGA group also profiled Chinese glioma patients into G1, G2, G3 groups based on gene expression, which matched the TCGA subtyping well [4]. However, the application of transcriptome subtype is still limited in clinical application because RNA sequencing and bioinformatic analysis are time-consuming and expensive.

Many genomic and epigenetic biomarkers for gliomas have been found in situ or blood [5–7]. Specific biomarkers also favor their expression in particular subtypes. For example, OLIG2, one of the four critical transcriptional factors of glioma stem cells, has a high expression in proneural GBM, which associates with relatively poor prognosis and drug resistance [8, 9]. Besides, CD276, also known as B7H3, has been proven to be associated with progression and poor prognosis [10, 11]. Interestingly, in our previous report, CD276 was found co-expressed with stem genes in GSCs and favored its expression in midline gliomas [12]. Our previous in-silico analysis also revealed that it could influence survival and mediate the G1/S transition in myc-driven neuroblastoma [13]. Similar efforts were made in medulloblastoma [14], and four distinct molecular subgroups of WNT, sonic hedgehog, group 3, and group 4 could guide therapeutic strategies [15]. Many other vital oncogenes like EGFR, CDK4, MDM2, GLI, PDGFRA, MET and MYC, were also investigated [16], and gene panels were used in the molecular profiling of GBM [17]. However, efficacy and efficiency are far from satisfactory, so that new biomarkers and methods to distinguish subtypes more efficiently are imperative.

To this end, we employed random forest, a machine-learning algorithm developed by Breiman [18]. Random-forest algorithm was widely used in classification [19, 20]. The algorithm has the following characteristics which distinguish it from other machine learning algorithms: (1) its ability to extract features, (2) the robust performance on noisy data with highly correlated variables, and (3) its ability to reduce the effect of the curse of dimensionality, i.e., high dimensional data with small sample size [21]. These characteristics make the random forest classifier an appropriate choice for gene expression datasets [22]. Furthermore, with the development of the next-generation sequencing (NGS), random forest has already been used extensively in the biomedical field, such as neurology [23], cancer classification and even protein–protein interaction sites prediction [24].

The current study aims to establish a gene cluster that could distinguish different molecular subtypes suitable for clinical application. Gene cluster featured with hub genes of OLIG2 and CD276 from our analysis will be conducted to distinguish molecular profiles.

## Methods

### Data collection and processing

Clinical information and array expression data of TCGA-GBM and Gravendeel were downloaded from Gliovis (http://gliovis.bioinfo.cnio.es/). Clinical information and RNA-seq data of GSE84010 were downloaded from Gene Expression Omnibus (https://www.ncbi.nlm.nih.gov/geo/query/acc.cgi?acc=GSE84010). All data were normalized for following analysis. Samples whose molecular classification are not available were excluded.

**Fig. 1:**
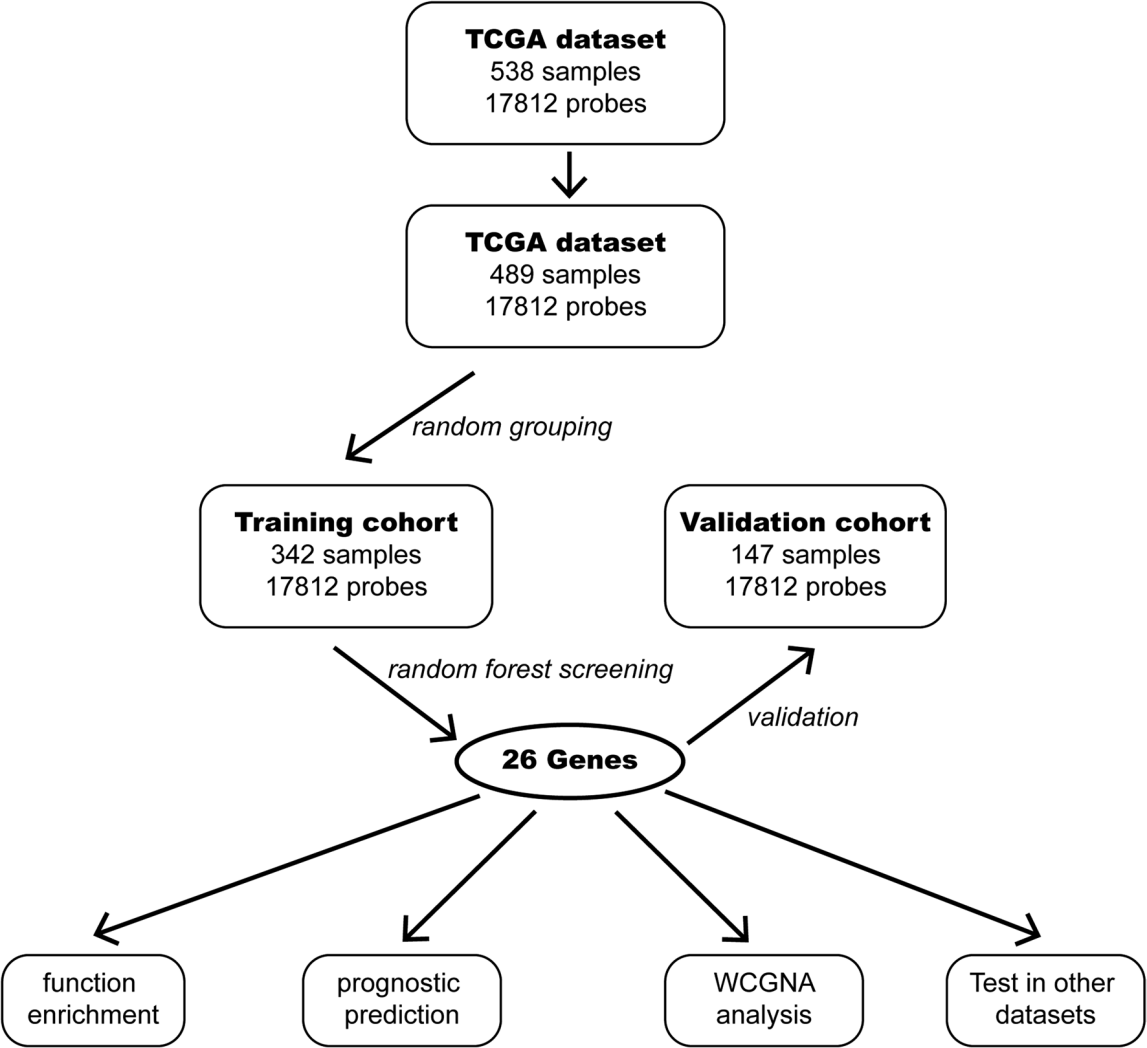
The flowchart of study design

The expression data of TCGA were assigned into two cohorts randomly for training and validation. Moreover, Gravandeel’s and GSE84010 datasets were used for the random forest model test. The flowchart of this study is shown in Fig. 1, and the baseline of the two cohorts was tabulated in Table 1. For further machine learning, all expression data was normalized.

**Table 1:** The baseline of training cohort and test cohort

Characteristic	Training cohort	Test cohort
Age, year		
Median	59.2	60.1
Range	10.9–89.3	25.2–86.6
Age, no. (%)		
< 60 year	52.9	49
≥ 60 year	46.2	49
NA	0	2
Sex, no. (%)		
Male	59.9	61.2
Female	38.9	35.4
NA	1.2	3.4
Primary or secondary, no. (%)		
Primary	94.4	93.2
Secondary	3.5	2.0
Recurrent	1.2	2.7
NA	0.8	2.0
IDH status, no. (%)		
Wild type	68.7	70.7
Mutant	6.1	4.1
NA	25.1	25.2
Subtype, no. (%)		
Classical	27.2	25.9
Mesenchymal	28.9	32.7
Neural	16.4	19
Proneural	27.5	32.7

There is no significant difference of the population baseline between the training cohort and test cohort

Data analysis was conducted using R 3.6.3 (R Core Team, 2020). A random number table generated by R 3.6.3 randomly assigned 70% of the patients to the training cohort (n = 342) and 30% to the validation cohort (n = 147).

### Immune infiltration analysis

The immune infiltration analysis was conducted by ImmuCellAI, a website tool (http://bioinfo.life.hust.edu.cn/ImmuCellAI#!/). ImmuCellAI can predict the abundance of 24 immune cell types in samples through a gene set signature-based method. The difference of immune cell infiltration in diverse groups was analyzed with the Immune cell abundance in groups [25, 26].

### Random forest training

Random forest was trained with R packages “randomForest”. The expression profiles were set as input, and the expression subtypes were set as labels. The number of decision trees (ntree) was set as 3000, and the max features (mtry) were set as 3. After every training, the input genes were ordered according to their importance, reflected by mean decreased accuracy and Gini. Genes whose mean decreased accuracy and Gini lower than OLIG2 and CD276 are excluded from the candidate genes until all genes were not lower than OLIG2 and CD276 in the form of mean decreased accuracy and Gini.

### Principal component analysis

Principal component analysis (PCA) based on the transcription matrix was performed using the R package “ggbiplot” (https://github.com/vqv/ggbiplot), and every gene is displayed in the coordinates with arrows from the origin.

### WGCNA construction and key module identification

The TCGA expression data profile was used for network generation by the R package WGCNA [26]. Initially, correlation of gene adjacency was conducted using a power function. Afterward, the modules were generated by the hierarchical average linkage clustering approach. The modules were assigned to different colors for visualization.

### Functional enrichment analysis

The functional enrichment analysis is performed with the R package “Clusterprofiler” [27]. We conducted the enrichment of genes in 3 modules we conducted from the PCA analysis, respectively and altogether. The results of the enriched terms of the Gene Ontology (GO), Kyoto Encyclopedia of Genes and Genomes (KEGG), and Disease Oncology (DO) pathways and were generated after running ClusterProfiler. The adjusted P-value < 0.05 was chosen as the threshold for identifying significant GO terms and pathways.

### Protein–protein interaction

The protein–protein interaction (PPI) data were downloaded from String (protein–protein interaction). Furthermore, the web plot was performed by the R package “igraph” (https://github.com/igraph).

### Prognostic prediction model construction

In order to find the predictive value of the individual genes, the proportional hazards (Cox) model was conducted. The results were summarized in forest plots and 2-dimension plots to show the risk factor for both PFS and OS. Calculations and graphing were conducted with the “survminer” and “ggforest” packages in R (https://cran.r-project.org/web/packages/survminer/, https://cran.r-project.org/web/packages/ggforest/).

To explore the prognosis predictive significance of the gene cluster, we conducted the most minor absolute shrinkage and selection operator (LASSO) with the “glmnet” package in R (https://cran.r-project.org/web/packages/glmnet/). LASSO calculated the risk score based on the gene expression level and regression coefficients.

## Results

### OLIG2 and CD276 share a mutually exclusive expression in gliomas

**Fig. 2:**
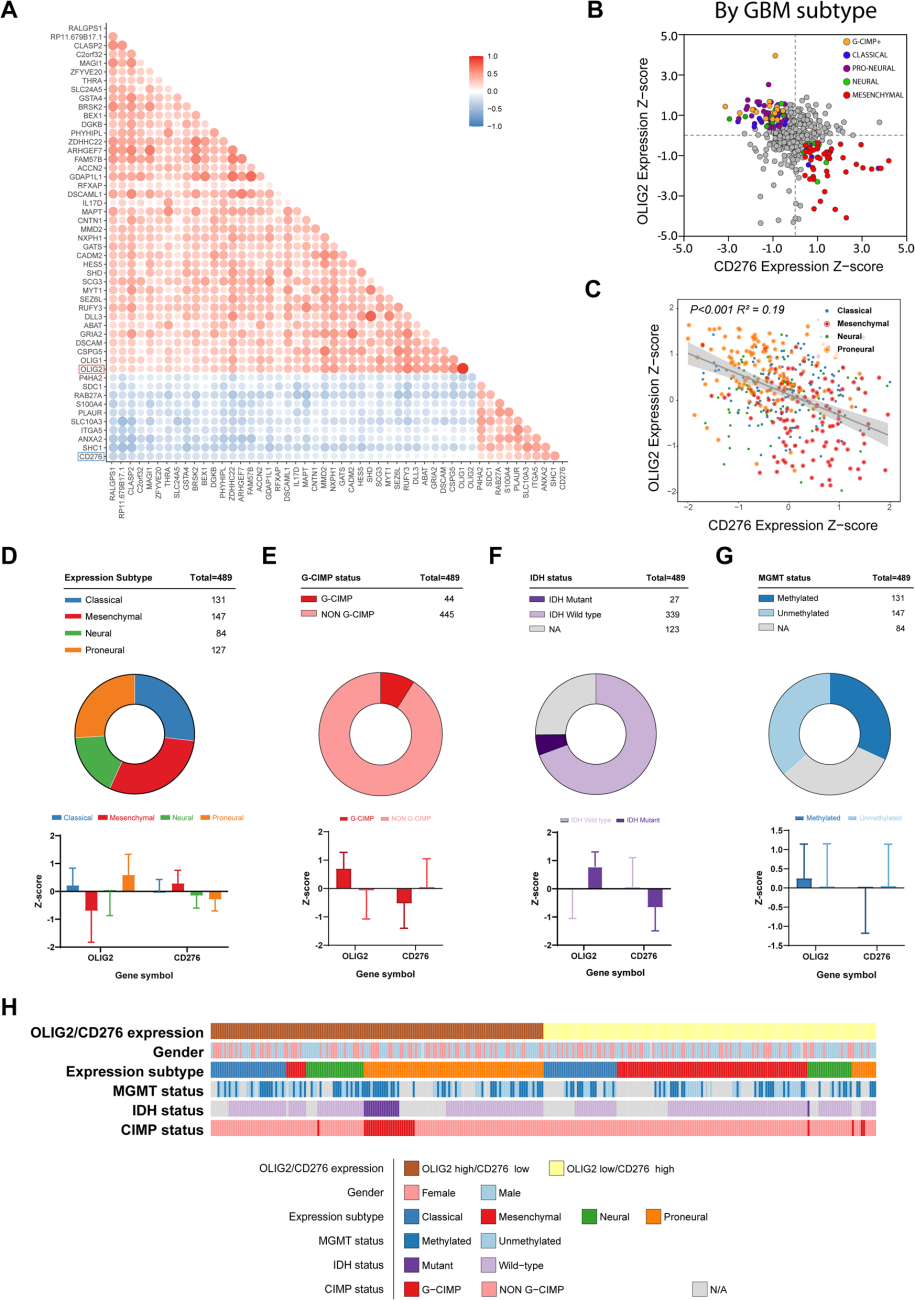
The exclusive expression correlation of OLIG2 and CD276 in different GBM subtypes. The expression of OLIG2 and CD276 is negatively correlated in TCGA GBM dataset (**A**). OLIG2 expression is high in proneural subtypes, while CD276 in mesenchymal (**B**–**D**). In GBM with G-CIMP status, IDH mutation status and MGMT methylation status, OLIG2 is highly expressed and CD276 shared exclusive expression pattern (**E**–**G**). The full view of the correlation of OLIG2/CD276 expression and other phenotypes is shown (**G**)

The bioinformatic analysis showed that OLIG2 and CD276 were negatively correlated (Pearson R = − 0.38, Spearman R = − 0.36, Fig. 2A) in TCGA GBM dataset (n = 489). The expression of OLIG2 and CD276 varied in different subtypes (Fig. 2B), and the mutually exclusive expression was subtype-depended (Fig. 2C), as CD276 favored in mesenchymal and OLIG2 in proneural subtypes (Fig. 2D). The regression analysis showed that the expression panel of CD276 and OLIG2 are in the opposite tendency (Radj2 = 0.19, P < 0.0001, Fig. 2C). Given G-CIMP status, OLIG2 is highly expressed in GBM with G-CIMP, while CD276 is high in GBM without G-CIMP (Fig. 2E). This contrary of CD276 and OLIG2 could also be observed in GBM with different IDH mutation status and MGMT methylation (Fig. 2F, G). All these data showed that OLIG2 and CD276 could share an exclusive expression pattern in GBM.

Since CD276 is an immune costimulatory molecule, we considered the association between the immune infiltration status and OLIG2/CD276 expression. We extracted OLIG2^hi^/CD276^lo^ and OLIG2^lo^/CD276^hi^ groups from the dataset according to the expression of OLIG2 and CD276 (the cutoff is mean expression). In comparing OLIG2^hi^/CD276^lo^ and OLIG2^lo^/CD276^hi^, immune infiltration score differs, especially in CD4 naïve cells, cytotoxic cells, Th1 cells, central memory cells, macrophage cells, neutrophil cells, Gamma delta cells, and infiltration score (Additional file 1: Fig. S1A). Moreover, gender, and MGMT status were not related to OLIG2/CD276 expression while IDH mutant and G-CIMP positive mainly belonged to OLIG2^hi^/CD276^lo^ group of GBM (Fig. 2H).

### PCA and WGCNA algorithm-generated gene clusters based on OLIG2 and CD276

**Fig. 3:**
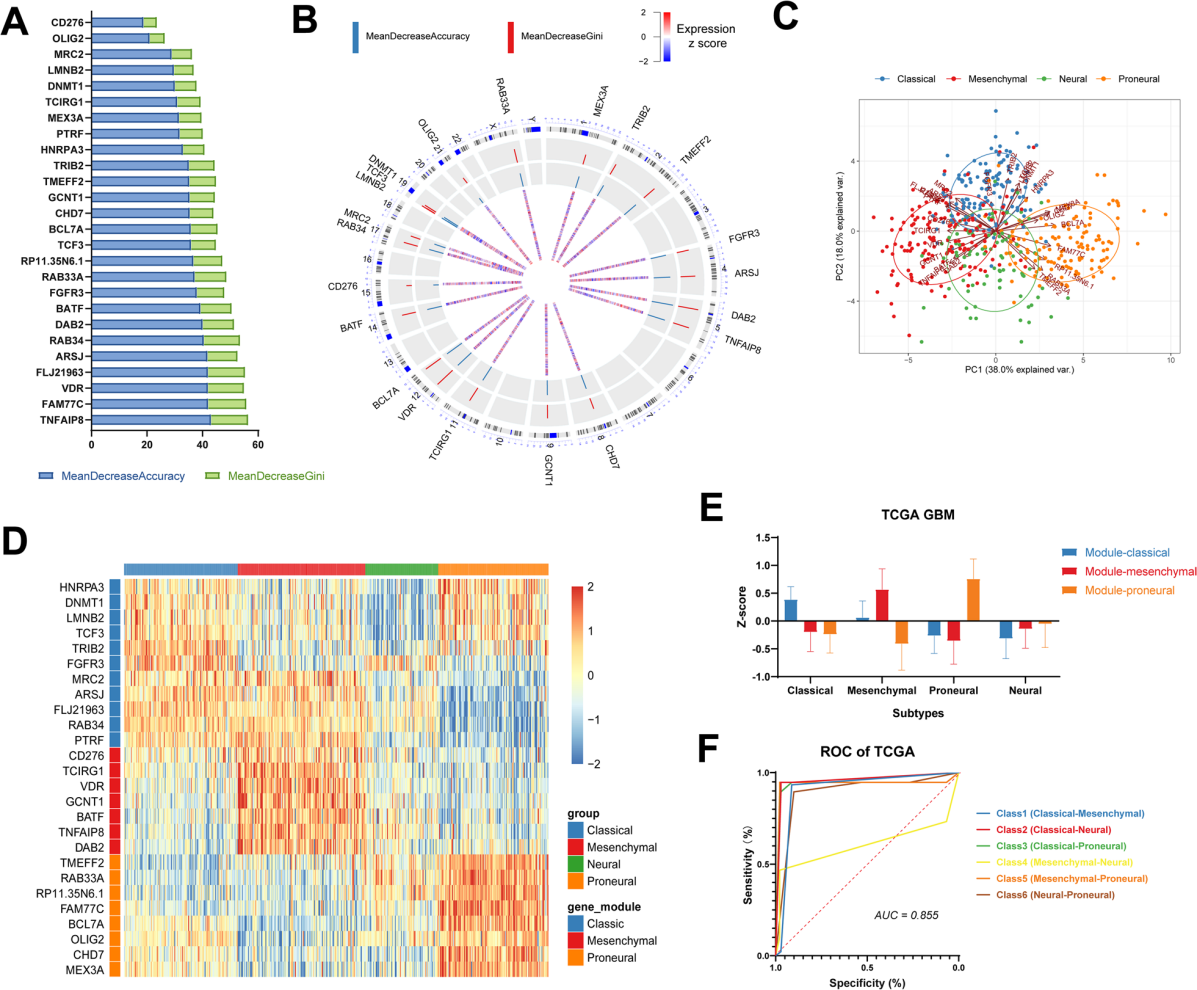
Gene clusters based on OLIG2 and CD276 generated by PCA analysis. 26 genes are obtained by random forest algorithm according to the expression subtypes (**A**). The full view of the locations of genes on chromatin is shown (**B**). PCA analysis revealed GBM subtypes can be identified clearly (**C**). Gene expression of three modules (module-classic, module-mesenchymal, and module-proneural) generated by PCA are shown in heatmap (**D**). Three gene modules expressed differently in four subtypes (**E**). ROC curve of the random forest algorithm for subtype classification is shown and the AUC reaches 0.855 (**F**)

Twenty-six genes were obtained by mean decreased accuracy and Gini with random forest algorithm under supervision according to the expression subtypes (Fig. 3A, B). PCA analysis was further performed to have visual sight of the expression of the genes in four subtypes. The 26 genes could distinguish the Verhaak’s subtypes well and be labeled with three modules (Module-Classic, Module-Mesenchymal, Module-Proneural) according to similar gene expression patterns. In detail, genes mainly positively reflected by principal component 1 (PC2 >|PC1|) were labeled Module-Classic. Moreover, genes whose PC1 >|PC2| and PC1 < − |PC2| were labeled as Module-Proneural and Module-Mesenchymal respectively (Fig. 3C). Genes in Module-Classic, Module-Mesenchymal, and Module-Proneural have higher expression levels in these modules than those in other modules (Fig. 3D), respectively. In detail, OLIG2 belonged to Module-Proneural and CD276 belonged to Module-Mesenchymal, consistent with the previous results. A column plot was displayed to show the expression of gene modules in 4 subtypes (Fig. 3E).

Furthermore, ROC was displayed to evaluate the efficiency of the random forest algorithm for subtype classification. Moreover, the AUC of all classes except mesenchymal versus neural is relatively high (AUC = 0.855, Fig. 3F). Specifically, the ability of the gene cluster to distinguish mesenchymal and proneural was excellent with an AUC value of 0.92. It is noteworthy that the proneural and mesenchymal subtype of GBM can be distinguished well by the gene cluster.

**Fig. 4:**
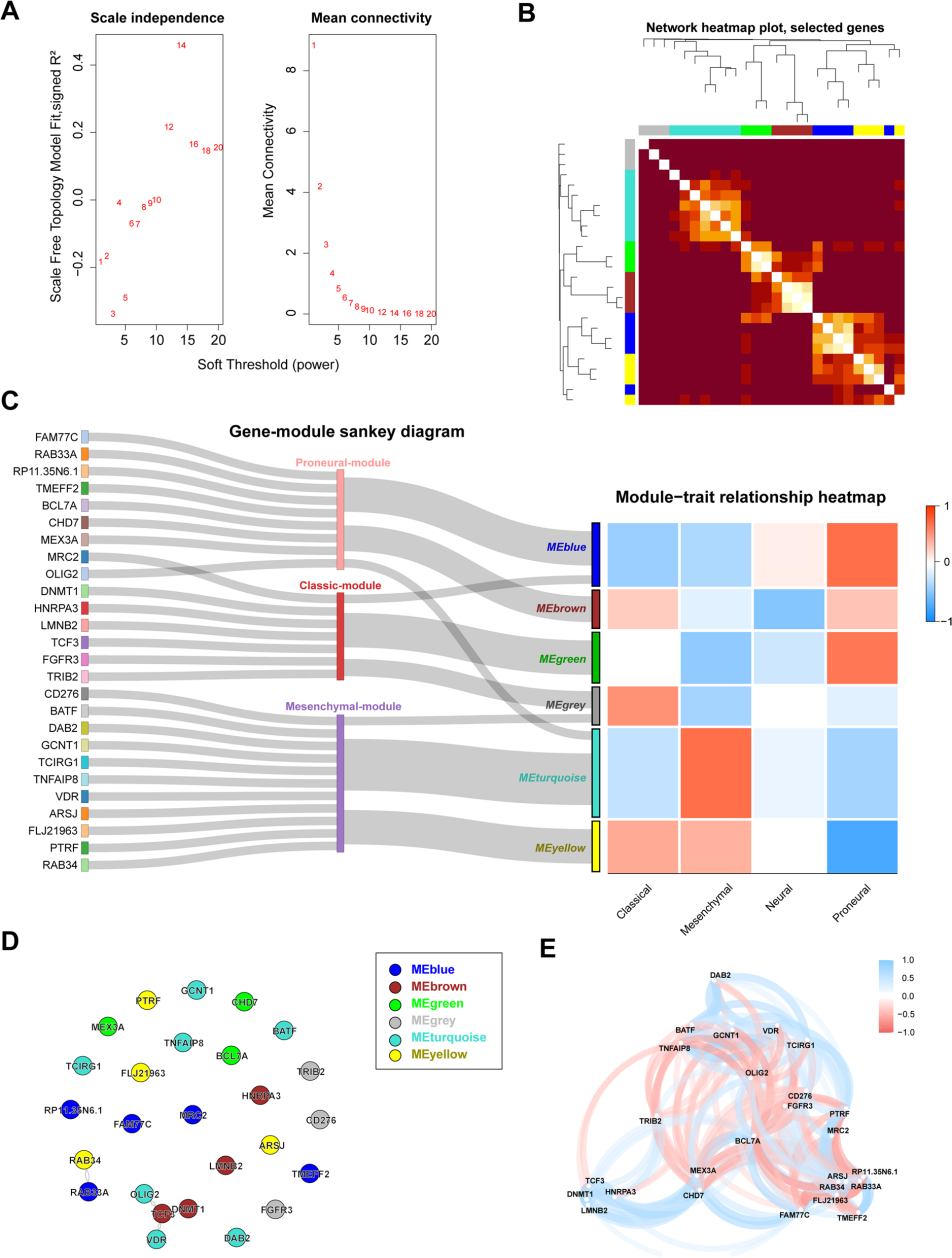
Gene clusters based on OLIG2 and CD276 generated by WCGNA algorithm. The soft threshold with corresponding scale free topology model fit and mean connection is set as 8 (**A**). TOM heatmap shows good cohesion of six modules generated by WCGNA algorithm (**B**). The Sankey diagram reveals existed correspondence between the two kinds of modules generated by PCA and WCGNA algorithm (**C**). RAB33A and RAB34 were exclusively expressed in mesenchymal and proneural GBM (**D**). Protein–protein network shows interaction among the gene clusters

The WCGNA algorithm was performed to match the co-expression network with the three principal component modules to validate the random forest algorithm. A soft threshold was set as 8, considering of the model’s accuracy and the computational expense (Fig. 4A). The gene clusters were grouped into six modules altogether (Fig. 4B). Further, the Sankey diagram was made to match the PCA analysis and WCGNA, and there existed correspondence between the two kinds of modules. Genes in MEblue and MEbrown belonged to the Proneural module, in high expression in proneural GBM indeed and in low expression in mesenchymal GBM. On the opposite, the vast majority of MEturquoise and the whole MEyellow belonged to the Mesenchymal module, highly expressed in mesenchymal GBM and lowly expressed in proneural GBM (Fig. 4C). The protein–protein interaction network was analyzed based on the modules to show the interaction among the gene clusters. As a result, only six genes in clusters (RAB34, RAB33A, OLIG2, VDR, TCF, and DNMT1) were found interactions by biochemistry experiments. RAB33A and RAB34 were Ras-related genes, the former was in MEblue, and the latter was in MEyellow. The two proteins were exclusively expressed in mesenchymal and proneural GBM (Fig. 4D). The network plot was displayed according to the correlation of the gene cluster, which matched the WGCNA results and PPI network very well (Fig. 4E).

### Gene clusters based on OLIG2 and CD276 could be validated in independent datasets

**Fig. 5:**
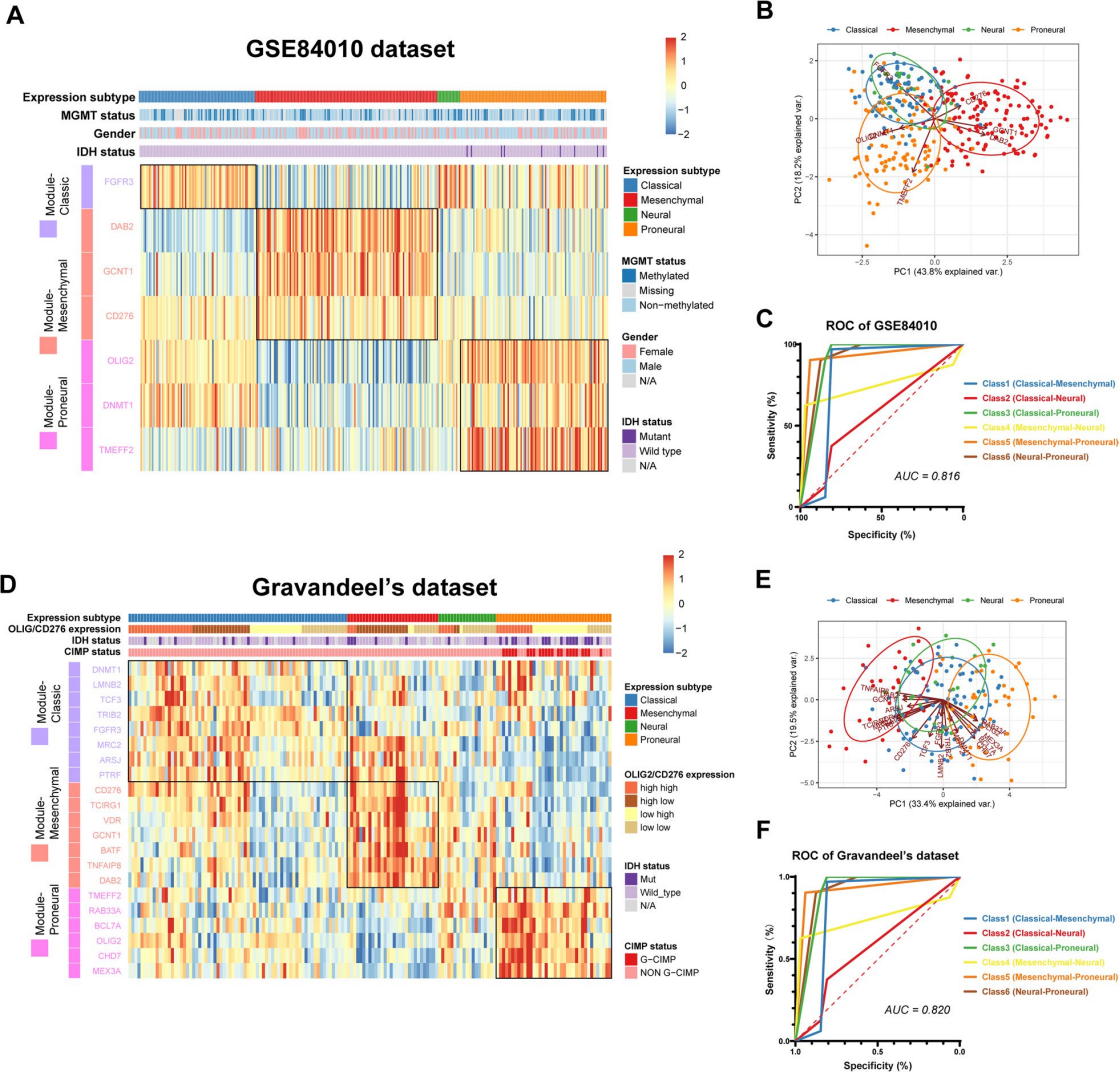
Validation of gene clusters in GSE84010 and Gravandeel’s GBM datasets. Heatmap shows good classifying ability of gene clusters in two independent datasets (**A**, **D**). PCA analysis reveals gene clusters could distinguish mesenchymal and proneural subtypes perfectly, but less distinguishable from classic and neural subtypes (**B**, **E**). ROC of random forest classification model reveals good efficacy (**C**, **F**) (AUC = 0.816 in the GSE84010 dataset, AUC = 0.820 in Gravandeel’s dataset)

GSE84010 and Gravandeel’s GBM datasets were used to test classifying efficacy. 7 of 26 genes are included in the GSE84010 dataset and 21 of 26 genes in Gravandeel’s dataset (Fig. 5A, D). ROC analysis showed the validation results on GSE84010 and Gravandeel’s datasets (Fig. 5C, F). The AUC of the random forest algorithm is still ideal (0.816 in the GSE84010 dataset, 0.820 in Gravandeel’s dataset). In detail, AUCs for mesenchymal-proneural classification in these two datasets exceeded 0.9, indicating the excellent efficacy of the gene cluster to distinguish mesenchymal and proneural subtypes. This feature was also displayed in the PCA plot, in which orange circle (referring to proneural subtypes) and red circle (referring to mesenchymal subtypes) were well distinguished (Fig. 5B, E). In general, the gene cluster showed good efficacy of the four expression subtypes generally.

### Gene clusters could be functionally enriched in DNA elements and T cell associated pathways

**Fig. 6:**
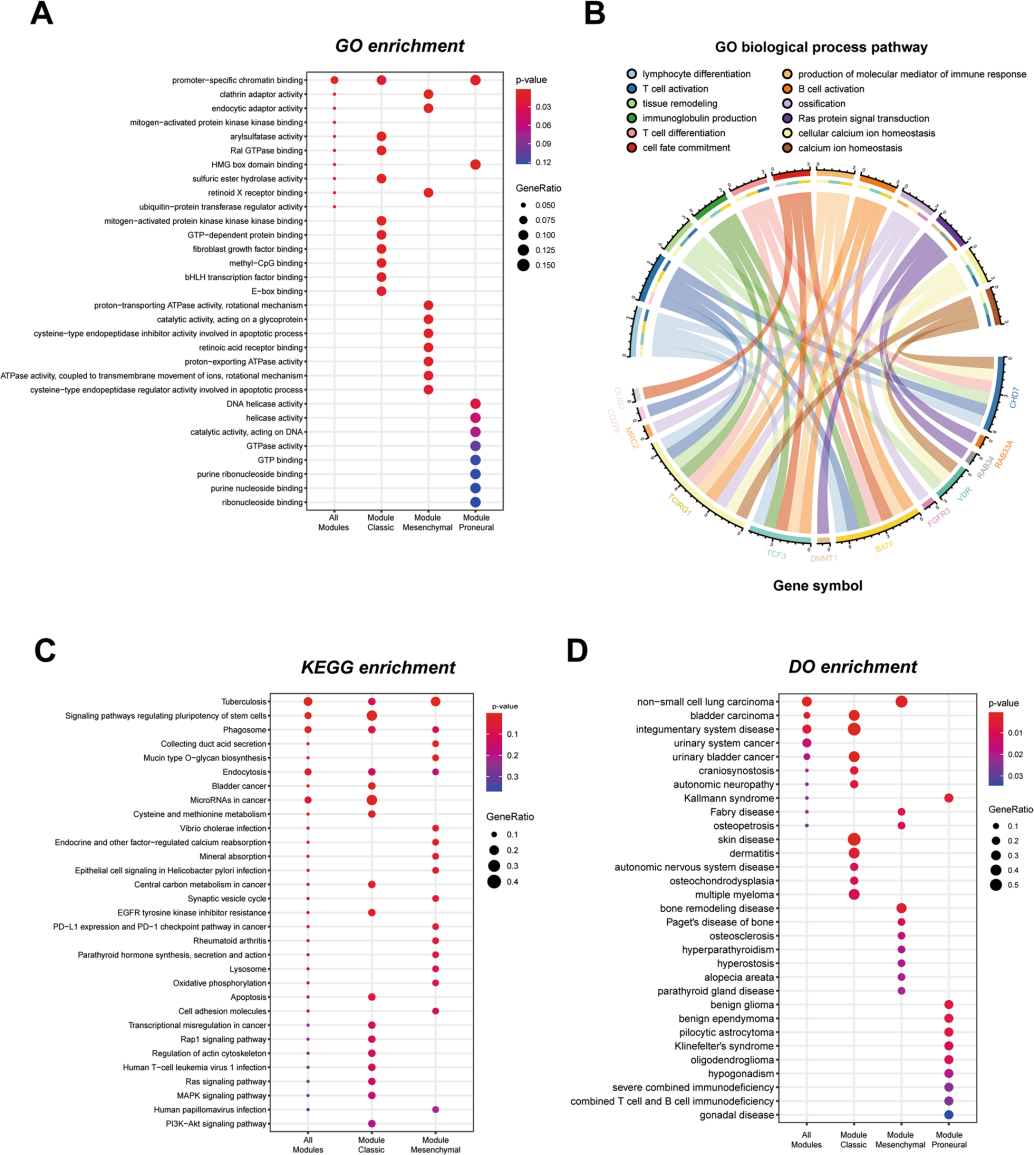
Functional enrichments of gene clusters. GO pathway enrichment revealed both classic and mesenchymal modules are enriched in DNA elements related pathway (**A**). GO biological process shows lymphocyte differentiation and T cell activation associated with four genes in the cluster (**B**). KEGG pathways reveals that signaling regulating pluripotency of stem cells is enriched (**C**). In DO database, genes in the cluster have a close connection with cancers (**D**)

To further explore the function of the 26-gene clusters, the GO, KEGG and DO database functional enrichment was performed for genes in different modules [28–30]. GO pathway enrichment revealed that both classic and mesenchymal modules are enriched in the promoter-specific chromatin binding pathway (Fig. 6A). Most enriched pathways from GO pathway analysis correlated with the DNA elements that regulate genes’ expression, such as promoter-specific chromatin binding, methyl-CpG binding, E-box binding, and catalytic activity, acting on DNA. Notably, GO biological process terms of lymphocyte differentiation and T cell activation was associated with four genes in the cluster, indicating the immune activity occupied an essential position in GBM as shown in the chord plot (Fig. 6B). KEGG database enriched pathways as signaling regulating pluripotency of stem cells and microRNAs in cancer indicating that genes in cluster associate with functions of pluripotency regulation (Fig. 6C). In the DO database, genes in the cluster were found to closely connect with cancers in other systems like non-small cell lung carcinoma, bladder carcinoma, and integumentary system disease (Fig. 6D). Furthermore, we found that genes in classic modules are also related to other common epithelial diseases like skin disease and dermatitis, and module-mesenchymal genes are associated with tumors originating from mesenchymal tissues, such as non-small cell lung carcinoma.

### Five genes in the gene clusters could construct a survival prediction model

**Fig. 7:**
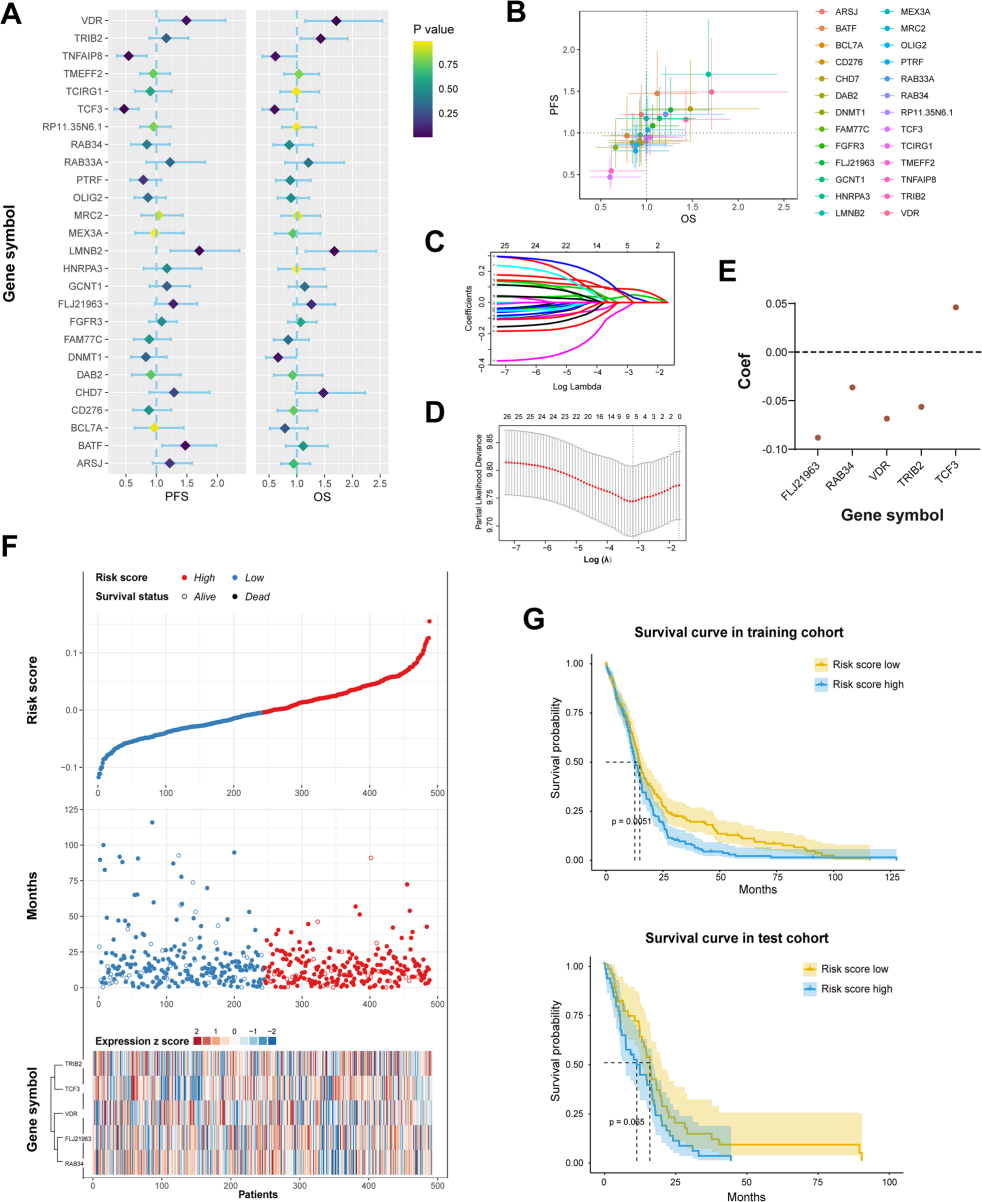
Survival prediction model based on genes in clusters. Multivariate Cox regression analysis revealed the association of 26 genes with OS and PFS (**A**). The predictive value of genes in cluster for OS and PFS is shown in 2-dimension plot (**B**). A signature of five genes is obtained by LASSO regression algorithm (**C**, **D**). The coefficients of five genes in the signature is shown (**E**). The full view of the risk score and the survival status based on five genes signature (**F**). The survival prediction model is tested in training cohort and test cohort (**G**)

Prognosis varied in different GBM subtypes (Additional file 2). In order to predict prognosis based on our gene panel, we conducted multivariate Cox regression analysis and revealed that VDR (HR for OS, 1.71; 95% CI, 1.15–2.54; p = 0.008; HR for PFS, 1.49; 95% CI, 1.04–2.13; p = 0.029), LMNB2 (HR for OS, 1.68; 95% CI, 1.16–2.43; p = 0.006; HR for PFS, 1.70; 95% CI, 1.23–2.36; p = 0.001), TCF3 (HR for OS, 0.60; 95% CI, 0.38–0.94; p = 0.027; HR for PFS, 0.47; 95% CI, 0.31–0.71; p < 0.001) and TNFAIP8 (HR for OS, 0.61; 95% CI, 0.38–1.00; p = 0.050; HR for PFS, 0.54; 95% CI, 0.35–0.84; p = 0.006) as the independent factors for both of OS and PFS (Fig. 7A).

Furthermore, a two-dimension plot was used to visualize the predictive value of genes in the cluster for OS and PFS. The closer the plot to the top right, the better prognostic the genes indicated (Fig. 7B). Through the LASSO regression algorithm, a signature of five genes was finally obtained (Fig. 7C, D). Only TCF3 expression negatively correlated with risk score (Fig. 7E). As shown in Fig. 7F, patients in the low risk-score group (Blue) benefit from longer survival time than those in the high risk-score group (Red). The prognosis of the low risk-score group is better than that of the high risk-score group in the training cohort and test cohort (Fig. 7G).

## Discussion

In addition to Verhaak’s classification, there are many attempts on gliomas classification. Karsy et al. found the subtypes were linked with some biomarkers, such as PDGFRA with proneural, NF1 with mesenchymal, ATRX mutation with astrocytomas, and pTERT mutation with oligdendrocytomas [31]. Wang et al. classified GBM into two types based on lineage markers, like type 2 shared oligodendrocyte differentiation and better prognosis [32]. Using a Random forest algorithm, Crisman et al. simplified Verhaak’s 840 total genes into 48 genes [33]. The accuracy of this approach ranged from 81.48% to 93.86%. Wang et al. applied 50-gene signatures to classify IDH wildtype GBM into three subtypes (mesenchymal, proneural, and classical) [34]. There was an improvement in the concordance from 77 to 93%.

Compared to the previous studies, our study found that the random forest algorithm performs efficiently in the subtype classification based on CD276 and OLIG2. The accuracy of our approach reaches 100% on the training set and 83.7% on the test set. The efficacy of the classification approach is high, given that our random-forest derived panel consists of twenty-six genes, half of the previous two studies [30, 31].

We concentrated on two genes (OLIG2 and CD276), both of which favored expression in proneural and mesenchymal expression subtypes. Therefore, we suppose that the two biomarkers, OLIG2 and CD276, represent two tendencies or subtypes of GBM, resulting in different expression profiles and prognosis status. Thus, through the identification of the two biomarkers, we can predict prognosis. Since these genes are potential biomarkers for some unique subtypes, target drugs can be administrated accordingly.

In the process of WGCNA analysis, we found some contradiction with the results of PCA analysis. In detail, OLIG2 belonged to Proneural-module and MEturquoise, which is in high expression in mesenchymal but in low expression in proneural. The bias of the model is not elusive to explain this mismatch. The mesenchymal-proneural transition might result in this phenomenon, based on the evidence that increased OLIG2 expression is considered a biomarker of mesenchymal-to-proneural transition (PMT). We also hypothesize that OLIG2 represented the expression subtype and might be a driver gene of the biological transition process. Loss of OLIG2 function in GSCs resulted in mesenchymal transformation, which indicates OLIG2 plays a vital role in PMT [35]. It is known that the Proneural subtype correlates with IDH mutated GBM. So, OLIG2 driven gliomas might have a relatively better prognosis, which is also demonstrated in this study. In the meantime, CD276 could be down-regulated in IDH mutated gliomas, mainly caused by autophagy induced by 2-HG accumulation [36]. CD276 also seems to favor its expression in H3 mutated gliomas [37], which is exclusive to IDH mutation. The malignancy represented by CD276 seems correlated with the TGF-beta pathway [38]. These findings indicate that different driving gene clusters might dominate in different subtype gliomas, resulting in different biological behaviors, clinical features, and prognosis.

Also, there are some limitations in this study. The sample size of the training cohort is small. As a result, our random forest algorithm-generated fewer decision trees to avoid overfitting, which might miss some essential genes. Another drawback is that, we did not take inter-tumor and intra-tumor heterogeneity into consideration when applying the random forest algorithm. Moreover, in order to obtain a simplified gene panel, a small number of genes were obtained. The weak association of a small number of genes leads to the low significance of the functional enrichment analysis, which might miss critical pathways. Furthermore, we found it is difficult to distinguish the neural subtype from the classical one, which is confirmed in other validation studies. The previous study based on a similar random forest method also found it challenging to distinguish neural subtypes [35]. The phenomenon could be attributed to the feature of the neural subtype. Wang et al. argued that the neural subtype GBM is non-tumor-specific [35, 39]. Because our understanding of GBM is still limited, further research was needed for more precise classification.

In conclusion, the random forest algorithm is proved efficient in the multi-classification of GBM expression subtypes, which would pave the way for precision medicine. With the development of sequencing, the combination of machine learning and next generation sequencing is likely to play an essential role in the diagnosing and predicting of GBM.

## Supplementary Information

**Additional file 1**:** Fig. S1.** The immune infiltration score in CD276^hi^/OLIG2^lo^ and CD276^lo^/OLIG2^hi^ groups. (p-value: *, < 0.05; **, < 0.01; ***, < 0.001).

**Additional file 2**: **Fig. S2.** The survival curve of TCGA, GSE84010, and Gravandeel’s dataset according to the three-subtype classifications (Proneural, Mesenchymal, and Classical).

## Data Availability

The datasets used or analyzed in this study are available from the corresponding author.

## Abbreviations

AUC: Area under curve
CGGA: Chinese Glioma Genome Atlas
CI: Confidence interval
COX: Proportional hazards model
DO: Disease Ontology
GBM: Glioblastoma
GO: Gene Ontology
KEGG: Kyoto Encyclopedia of Genes and Genomes
LASSO: Least absolute shrinkage and selection operator
NGS: Next Generation Sequencing
PCA: Principal component analysis
PMT: Mesenchymal-to-proneural transition
PPI: Protein–protein interaction
ROC: Receiver operating characteristic curve
TCGA: The Cancer Genome Atlas
WGCNA: Weighted correlation network analysis
