# Supplementary figures and images for: Gene clusters based on OLIG2 and CD276 could distinguish molecular profiling in glioblastoma
Source: J Transl Med. 2021 Sep 26;19:404. doi: 10.1186/s12967-021-03083-y (PMC8474912; doi:10.1186/s12967-021-03083-y)

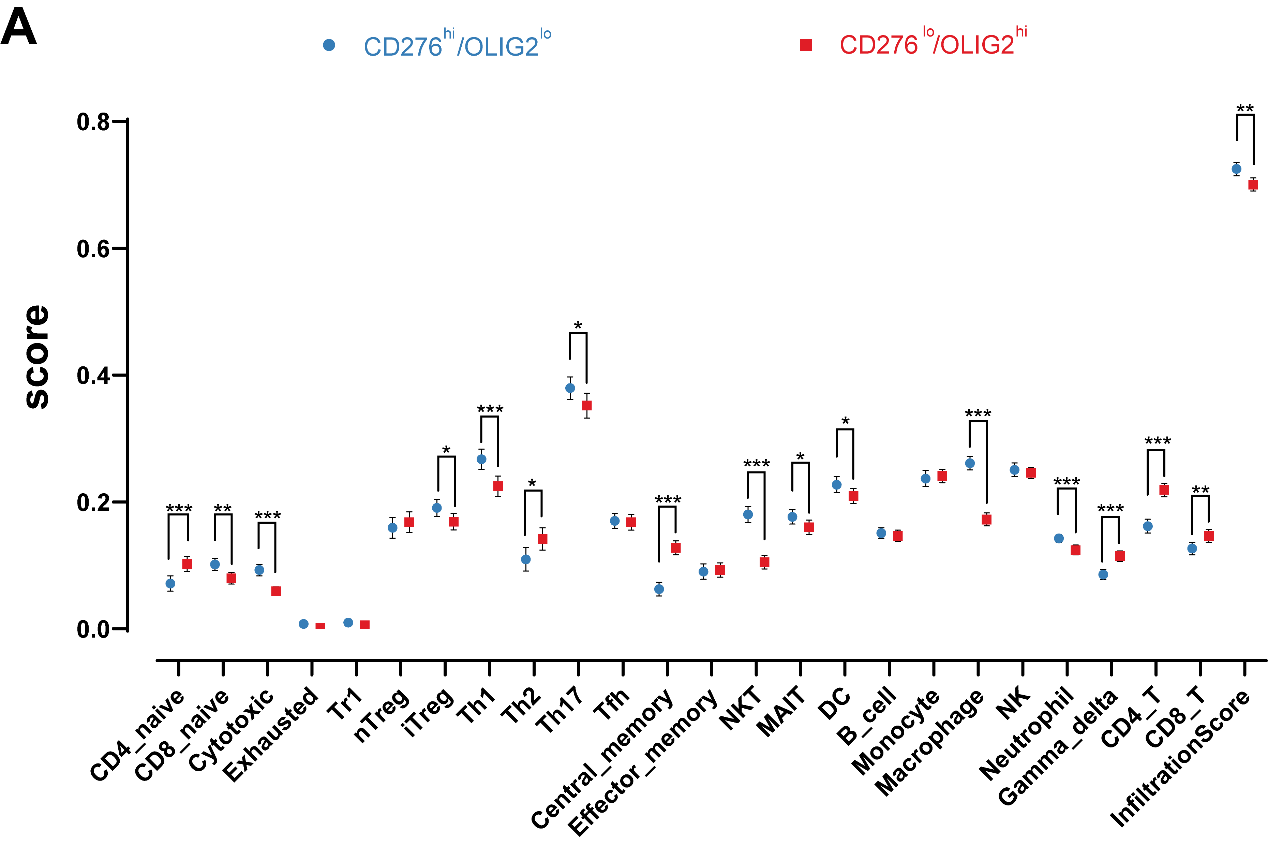


**Fig. S1:** The immune infiltration score in CD276hi/OLIG2lo and CD276lo/OLIG2hi groups. (p-value: *, < 0.05; **, < 0.01; ***, < 0.001).

Supplement: Supplementary file 1 — Additional file 1: Fig. S1. The immune infiltration score in CD276hi/OLIG2lo and CD276lo/OLIG2hi groups. (p-value: *, < 0.05; **, < 0.01; ***, < 0.001). [file 12967_2021_3083_MOESM1_ESM.docx]
