# Supplementary material for: Gene clusters based on OLIG2 and CD276 could distinguish molecular profiling in glioblastoma
Source: J Transl Med. 2021 Sep 26;19:404. doi: 10.1186/s12967-021-03083-y (PMC8474912; doi:10.1186/s12967-021-03083-y)
Supplement: Supplementary file 2 — Additional file 2: Fig. S2. The survival curve of TCGA, GSE84010, and Gravandeel’s dataset according to the three-subtype classifications (Proneural, Mesenchymal, and Classical). [file 12967_2021_3083_MOESM2_ESM.docx]

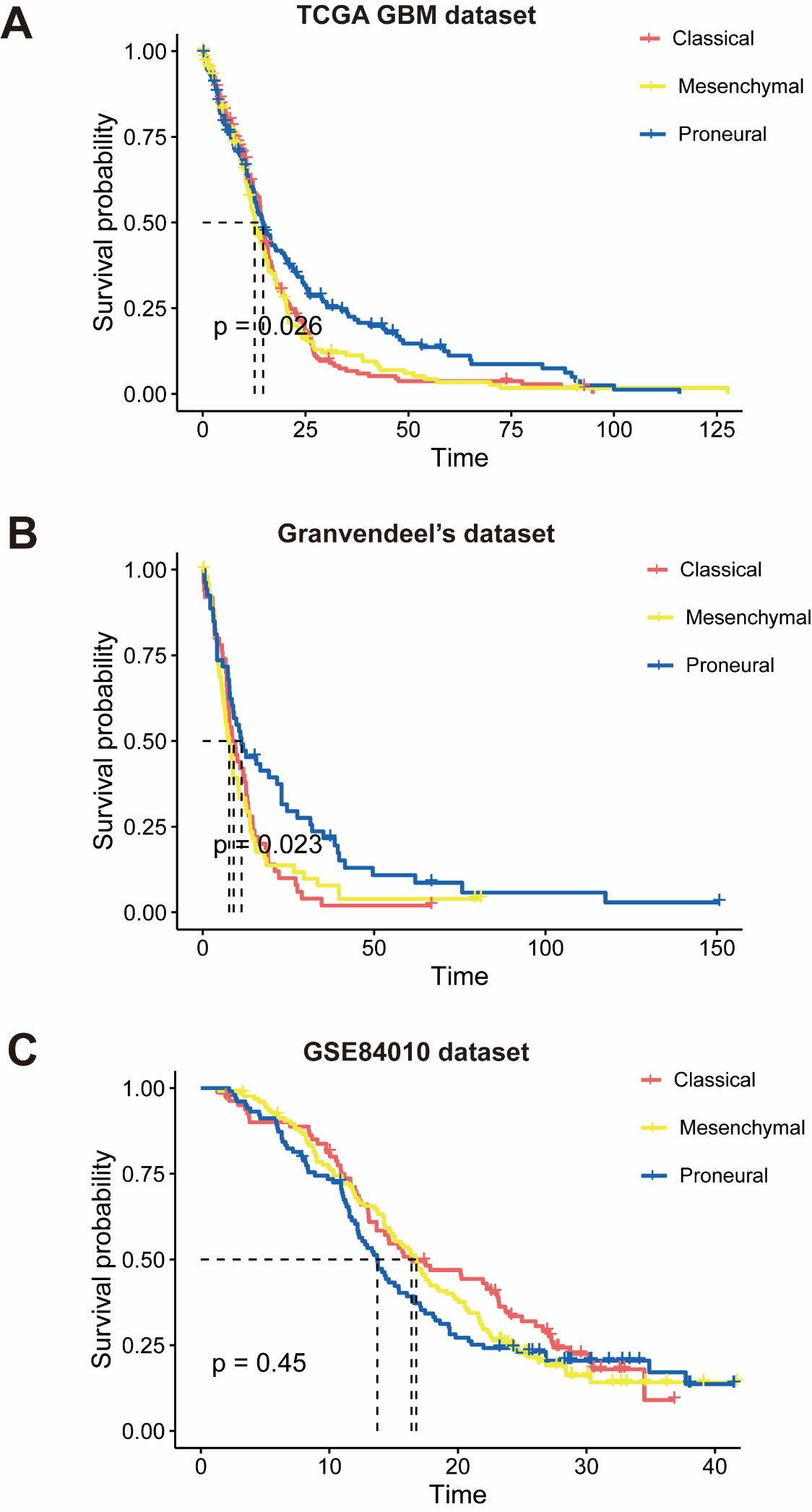
 **Fig. S2**: The survival curve of TCGA, GSE84010, and Gravandeel's dataset according to the three-subtype classifications (Proneural, Mesenchymal, and Classical).
